# Well-being in chronic pediatric inflammatory rheumatic diseases: the experience of a French healthcare network

**DOI:** 10.1186/s13023-023-02655-z

**Published:** 2023-03-07

**Authors:** Rita El Haddad, Khalil El Asmar, Chrystelle Hascoët, Linda Rossi-Semerano, Perrine Dusser

**Affiliations:** 1 Réseau Rhumatismes Inflammatoires Pédiatriques (RESRIP), Bourg-la-Reine, France; 2 Public Health Faculty, Lebanese University, Fanar, Lebanon; 3 Faculty of Medicine, Université Paris-Saclay, Université Paris Sud, Le Kremlin Bicêtre, France; 4 Paediatric Rheumatology Department, APHP, Bicêtre Hospital, Université Paris-Saclay, 94270 Le Kremlin-Bicêtre, France; 5 Centre de Référence des Maladies Auto-Inflammatoires et des Amyloses Inflammatoire (CEREMAIA), Le Kremlin Bicêtre, France

**Keywords:** Quality of life, Chronic inflammatory rheumatic diseases, Children, Adolescents, Healthcare network

## Abstract

**Objective:**

Current management of patients with pediatric rheumatic diseases (PRD) should aim at achieving the best possible well-being.

To identify sociodemographic/clinical characteristics, needed paramedical services and school accommodations associated with well-being in patients at inclusion in a French health network Réseau pour les Rhumatismes Inflammatoires Pédiatriques (RESRIP) that supports coordination of the patient’s health pathway. To evaluate the evolution of well-being over time in this patients benefiting from such support.

**Methods:**

Patients > 3 years old enrolled in RESRIP (2013–2020) were included. At enrollment, data were collected on sociodemographic/clinical characteristics, ongoing medications, and paramedical and educational actions to be implemented by RESRIP. Well-being during the last 6 months was reported with a standardized questionnaire at enrollment and every 6 months. A well-being score was calculated with scores ranging from 0 to 18, 18 corresponding to absolute well-being. Patients were followed up from inclusion until June 2020.

**Results:**

In total, 406 patients were included and followed up for 36 months on average: 205 juvenile idiopathic arthritis, 68 connective tissue diseases, 81 auto-inflammatory diseases and 52 other diseases. The well-being score did not differ between the groups and improved significantly, by 0.04 score units, every 6 months (95% confidence interval [0.03; 0.06]). At inclusion, use of homeopathy, need for implementation of hypnosis or psychological support, occupational therapy or for adjustment of school tests were associated with worse well-being score.

**Conclusion:**

Well-being seems associated more with the impact of chronic illness than the type of PRD underlining the importance of a comprehensive patient care.

**Supplementary Information:**

The online version contains supplementary material available at 10.1186/s13023-023-02655-z.

## Introduction

Chronic rheumatic diseases (CRDs) encompass a broad group of inflammatory disorders such as juvenile idiopathic arthritis (JIA), connective tissue diseases (CTDs) and auto-inflammatory diseases (AIDs) that result from innate immune dysregulation [1–3]. With the advent of biotherapies, the management of these CRDs has been revolutionized with improvements in both disease activity and well-being. Yet, adults and children with CRDs still report suboptimal well-being [4–8], particularly in relation to school, health care and public institutions [4, 8–16]. The current management of patients with CRDs should therefore not be limited to achieving clinical and biological remission, but should aim at achieving the best possible well-being especially as the indicators used by physicists to assess therapeutic efficacy are not always aligned with patient welfare.

The rarity of rheumatic diseases in children, their lack of awareness and delay in diagnosis, as well as the difficulty of setting up and coordinating a variety of trained health professionals practicing close to the patients' homes, prompted the creation of a dedicated health network. In this context, the first French health network for rare disease, RESRIP (RESeau pour les Rhumatismes inflammatoires pédiatriques), for children/adolescents with CRDs under 18 years old, was created in 2013. RESRIP's two main objectives are: 1- Coordination of the health pathway and 2- Support for health professionals. RESRIP is funded by the ARS (Regional Health Agency) Ile-De-France and allows to strengthen the city-hospital link for patients living in this particular region free of charge. Patients are referred to RESRIP by their referring physician if they are considered to be in need of support due to a serious illness, poor social conditions and/or a need for schooling. RESRIP acts as a link with the patient’s various medical, para-medical and social health professionals. It does not provide care but day-to-day support, particularly in terms of education. It establishes contact with schools or professional establishments to ensure that its pupils/students are properly integrated, and organizes multidisciplinary meetings with school nurses and/or doctors, as well as with teachers, to explain the pathology or resolve certain tensions or misunderstandings. Finally, RESRIP has a cross-sectional PTE program (www.resrip.fr) for children with CRDs and their families to help them manage their disease on a daily basis.

Understanding the factors associated with well-being seems therefore essential today. Thus, we first sought to identify the sociodemographic/clinical characteristics, medical and paramedical therapy needs and schooling accommodations associated with well-being in patients at inclusion in RESRIP. Secondly, we sought to evaluate the evolution of well-being over time in these patients benefiting from comprehensive care and daily support by RESRIP.

## Methodology

### Study population

In this longitudinal study, patients enrolled in the RESRIP health network between October 2013 and June 2020 were eligible for inclusion. Only patients who at inclusion were > 3 years old were considered to have a social activity and were included in the study. Patients could be enrolled at any time during their disease course. To be included in the RESRIP network, patients had to fulfill > 2 of the following 7 criteria: complex pathology, need for > 3 health professionals, association of ≥ 2 diseases, precarious social context, request for support from the patient and/or the family, transition to adult medicine, removal from an expert center.

At inclusion, during a face-to-face interview with a nurse of the RESRIP team, a standardized questionnaire was completed by the patients or their parents/guardians. This interview allows RESRIP team to know the patient and the family and understand their needs in order to set up targeted actions. RESRIP’s purpose is to facilitate the daily life of families by finding trained health professionals and setting up social and educational actions. It also aims to support the medical, paramedical and school professionals of these children/adolescents [17]. Actions to be implemented by RESRIP are then put in place. Every 6 months, the same questionnaire is completed face-to-face or by phone. For families who cannot be reached, the questionnaire is sent directly by mail and completed by the parents/guardians and/or the child (> 7 years old). Responses are stored in a medical file by using ICT-Chorus software certified in accordance to the RGPD law (HDS certification as well as ISO 27001).

The study was performed according to regulations of the local ethics committee. Informed consent was obtained from each patient before enrolment. Data were extracted and anonymized by using a numerical identifier.

### Measures

#### Diagnosis

At inclusion, the diagnosis was provided by the patient or parents/guardians and confirmed by the referring physician. According to the diagnosis, patients were divided into 4 major disease groups: JIA, CTD, AIDs and other diseases.

#### Sociodemographic characteristics

At the enrollment visit, data were collected on sex, age, date of disease onset and of disease diagnosis. According to the age at the time of inclusion patients were classified into 3 groups: preschooler (3–5 years old), middle schooler (6–11 years old) and adolescent (≥ 12 years old). Disease duration at the time of inclusion was calculated and grouped into 2 categories: < 1-year evolution and ≥ 1-year evolution. Information regarding patients’ number of school days’ absenteeism over the last 6 months were collected at EV and every 6 months. We then dichotomized school absenteeism into two groups: non-absenteeism (less than 12 days per 6 months) and absenteeism (12 days or more per 6 months) since 4 half days per month were considered as regulatory threshold of absenteeism as defined by the French national education system (https://www.education.gouv.fr).

#### Ongoing medications

Information on ongoing treatment was collected, including non-steroidal anti-inflammatory drugs (NSAIDs), glucocorticoids (GCs), analgesics, joint infiltration agents, disease-modifying anti-rheumatic drugs (DMARDs), biologic agents, colchicine, contraception and homeopathy.

#### Actions to be implemented by RESRIP

After the inclusion interview, information was collected on actions to be implemented by RESRIP both at the paramedical level (need for orthopedic devices, social assistance, occupational therapyhypnosis or related approaches for chronic pain, home care nurse, physical therapy, general practitioner, pediatrician, psychological support and diet monitoring) and school level (school planning, test accommodations, arrangement for school sports activity).

#### Well-being

Information regarding patients’ perception of their illness, social and family life, i.e. their “well-being” was collected at the enrollment visit and were self-reported every 6 months using a standardized questionnaire created by RESRIP that covered topics deemed relevant for monitoring the coordination of care and the actions set up or implemented by the health network at the social and school level. Topics included well-being at home, with friends and at school and physical health (using a radical scale ranging from “very satisfied” to “very dissatisfied”); fatigue (ranging from none to significant); nocturnal awakening (0, 1, 2, 3 or > 3); and physical health (0–10 scale) (Additional file 2: Appendix 1). A well-being score ranging from 0 to 18 (18 corresponding to the best well-being), was calculated by adding the score for all questions regarding physical and social life, each item ranging from 0 to 3 (Table 1). Although to the term « health-related quality of life» that is very specific to health is more commonly used, we chose the term « well-being» because well-being is a more meaningful measure for people, as it indicates how people perceive their lives from their own perspective, beyond morbidity, mortality and socioeconomic status. It includes overall judgments about life satisfaction and feelings such as well-being at home, at school and with friends [18]. We evaluated patients included in the RESRIP between October 2013 and June 2020; the follow-up period for each patient depended on the date of inclusion.

**Table 1:** Scoring of the items of the well-being questionnaire for children/adolescents with chronic inflammatory rheumatic diseases (n = 406)

Item	Score			
	0	1	2	3
Fatigue	Important	Moderate	Poor	None
Nocturnal awakening	≥ 3 times/night	Twice per night	Once per night	0 times per night
Physical health	Poor	Fair	Satisfactory	Very satisfactory
Life at school	Poor	Fair	Satisfactory	Very satisfactory
Life with friends	Poor	Fair	Satisfactory	Very satisfactory
Life with family	Poor	Fair	Satisfactory	Very satisfactory

#### Statistical analysis

Statistical analyses were performed with Stata 13.1. Descriptive parameters are reported with frequencies (percentage). The well-being score is reported with mean (standard deviation [SD]). Data were reshaped to a long format and the variable “month of follow-up” was created. Missing data were not replaced. Cronbach’s alpha was calculated to measure the internal consistency of the standardized questionnaire. Univariate analyses were performed with mixed linear models, and all covariates with *p* < 0.05 were included in the final multivariable model along with the variables month of follow-up (treated as a continuous variable), age at inclusion, sex and diagnosis to determine the association with well-being, and how it evolved over time. We estimated 95% confidence intervals (CIs). *p* < 0.05 was considered statistically significant.

## Results

In total, 406 of 465 children/adolescents registered in RESRIP were ≥ 3 years old and were included in this study. The average missing data per variable was 8.6% and ranged from 1.1% for duration of illness to 20.6% for father’s occupation. Regarding our outcome, well-being, 67 (16.5%) participants had no total well-being score at inclusion or at follow-up.

Two hundred and five patients were diagnosed with JIA (50.5%), 68 with CTDs (16.8%), 81 with AIDs (19.9%) and 52 with other diseases (12.8%) (Table 2). Girls represented 64% of the population. The demographic characteristics at the enrollment visit are in Table 2: almost half of the participants (49.8%) were > 12 years old and most had disease duration ≥ 1 year (61.7%). The mean follow-up period was 36 (0.30) months, with an average of 2.2 (1–7) follow-ups. In total, 209 (51.5%) patients were taking NSAIDs, 151 (37.2%) pulse or oral GCs, 157 (38.7%) DMARDs and 117 (28.8%) biologics (Table 3). At inclusion, 16% (52/320) of the children reported with school absenteeism.

**Table 2:** Sociodemographic characteristics of children/adolescents > 3 years old at the time of inclusion (n = 406)

	N (%)
*Diagnosis (n* = *406)*	
Juvenile idiopathic arthritis	205 (50.5)
Connective tissue diseases	68 (16.8)
Auto inflammatory diseases	81 (19.9)
Other	52 (12.8)
*Age at inclusion [years] (n* = *406)*	
Preschooler [3–5]	58 (14.3)
Middle schooler [6–11]	146 (35.9)
Adolescent [≥ 12]	202 (49.8)
*Sex (n* = *406)*	
Girls	260 (64.0)
Boys	146 (36.0)
*Disease duration (n* = *399)*	
< 1 year	153 (46.3)
≥ 1 year	246 (61.7)

**Table 3:** Ongoing treatments at inclusion in the RESRIP network by disease (n = 406)

	Total (n = 406)	JIA^a^ (n = 255)	CTD^b^ (n = 69)	AIDs^c^ (n = 80)	Other (n = 57)
NSAIDs^d^	209 (51.5)	138 (67.3)	18 (26.5)	44 (54.3)	9 (17.1)
Joint infiltration	40 (9.9)	40 (19.5)	0 (0)	0 (0)	0 (0)
Biologic therapy	117 (28.8)	86 (41.9)	3 (4.4)	19 (23.5)	9 (17.3)
Colchicine	41 (10.1)	1 (0.5)	2 (2.9)	36 (44.4)	2 (3.9)
DMARDs^e^	157 (38.7)	70 (34.2)	45 (66.2)	18 (22.2)	24 (46.1)
Glucocorticoids	151 (37.2)	51 (24.9)	46 (67.6)	28 (34.6)	26 (50.0)
Contraception	9 (2.2)	5 (2.4)	2 (2.9)	1 (1.2)	1 (1.9)
Homeopathy	9 (2.2)	5 (2.4)	0 (0)	2 (2.5)	2 (3.8)
Other treatment^f^	216 (53.2)	98 (47.8)	56 (82.3)	35 (43.2)	27 (51.9)

^a^Juvenile idiopathic arthritis

^b^Connective tissue disease

^c^Auto-inflammatory diseases

^d^Non-steroidal anti-inflammatory drugs

^e^Disease-modifying antirheumatic drugs

^f^Vitamins, proton pump inhibitor, hydroxychloroquine and other

### Actions to be implemented by RESRIP after the inclusion interview (Table 4)

**Table 4:** Actions implemented by RESRIP at the paramedical, social and educational levels, at the request of the patient and/or the referring doctor, at the time of inclusion (n = 406)

	Total (n = 406)	JIA (n = 256)	CTD (n = 68)	AIDs (n = 82)	Other (n = 55)
*School accommodation*					
Educational actions	54 (13.3)	27 (13.2)	10 (14.7)	9 (11.1)	8 (15.4)
Test accommodation	30 (7.4)	14 (6.8)	5 (7.4)	8 (9.9)	3 (5.8)
Arrangement for sports activity	153 (37.7)	72 (35.1)	31 (45.6)	34 (42.0)	16 (30.8)
*Health professionals*					
General practitioner	322 (79.3)	158 (77.1)	56 (82.4)	62 (76.5)	46 (88.5)
Pediatrician	77 (19.0)	53 (25.9)	9 (13.2)	6 (7.4)	9 (17.3)
Social assistant	47 (11.6)	14 (6.8)	15 (22.1)	9 (11.1)	9 (17.3)
Home care nurse	131 (32.3)	82 (40.0)	20 (29.4)	16 (19.8)	13 (25.0)
*Supportive care*					
Psychological support	90 (22.2)	37 (18.1)	13 (19.1)	24 (29.6)	16 (30.8)
Dietetic monitoring	41 (10.1)	12 (5.8)	16 (23.5)	6 (7.41)	7 (13.5)
Orthopedic devices	27 (6.6)	19 (9.3)	2 (2.9)	2 (2.5)	4 (7.7)
Occupational therapy	30 (7.4)	18 (8.8)	4 (5.9)	2 (2.5)	6 (11.5)
Hypnotherapy	52 (12.8)	21 (10.2)	6 (8.8)	14 (17.3)	11 (21.1)
Physical therapy	239 (58.9)	161 (78.5)	40 (58.8)	20 (24.7)	18 (34.6)

*JIA* juvenile idiopathic arthritis, *CTD* connective tissue disease, *AIDs* auto-inflammatory diseases

Various medical, paramedical and social actions were set up by RESRIP at the request of the patient and/or the family in agreement with the referring physician. Regarding paramedical care, 90 (22.2%) patients received psychological follow-up with a psychologist and 52 (12.8%) from hypnosis sessions. School facilities were set up for 54 (13.3%) patients, facilities for national examinations for 30 (7.4%) and facilities for physical and sports education set up for 153 (37.7%).

### Well-being score (Table 5)

**Table 5:** Well-being score at the time of inclusion in the RESRIP by disease group (n = 220)

Diagnosis	JIA (n = 111)	CTD (n = 34)	AIDs (n = 40)	Other (n = 35)
Well-being score	11.82 (0.4)	11.70 (0.6)	10.07 (0.6)	12.11 (0.7)

Data are mean (SD)

*JIA* juvenile idiopathic arthritis, *CTD* connective tissue disease, *AIDs* auto-inflammatory diseases

The questionnaire had internal consistency, with a Cronbach's alpha of 0.77. At inclusion, AIDs patients had the worst mean (SD) well-being score (10.07 [0.6]) followed by CTD (11.70 [0.6]) and JIA patients (11.82 [0.4]). The mean well-being score for patients at inclusion was 11.53 [0.2].

### Patient characteristics associated with well-being score at inclusion (Table 6)

**Table 6:** Association between medico-socio-demographic characteristics and well-being score for the RESRIP population at inclusion

	Coefficient	95% CI	*p*-value
Month of follow-up	0.04	[0.03; 0.06]	< 0.001
Age at inclusion			
Preschooler	–	–	–
Middle schooler	0.13	[− 0.77; 1.04]	0771
Adolescent	0.04	[− 0.96; 0.87]	0.917
Sex			
Male	–	–	–
Female	0.44	[− 0.19; 1.08]	0.169
Diagnosis			
JIA^a^	–	–	–
CTD^b^	− 0.36	[− 1.24; 0.51]	0.414
AIDs^c^	− 0.79	[− 1.77; 0.17]	0.109
Other	0.52	[− 0.46; 1.51]	0.296
Disease duration > 1 year	− 0.01	[− 0.66; 0.64]	0.982
Educational actions	− 0.69	[− 1.57; 0.18]	0.120
Test accommodation	− 1.55	[− 3.04; − 0.06]	0.041
Occupational therapy	− 1.26	[− 2.41; − 1.22]	0.030
Hypnotherapy	− 1.29	[− 2.22; − 0.36]	0.006
Psychological support	− 1.45	[− 2.21; − 0.70]	< 0.001
Colchicine	− 0.61	[1.91; − 0.68]	0.352
Contraception	− 2.01	[− 4.04; 0.02]	0.053
Homeopathy	− 1.96	[− 3.70; − 0.22]	0.026
DMARDs^d^	0.51	[0.13; − 1.14]	0.121
Joint infiltration	1.02	[− 0.07; 2.11]	0.066

^a^Juvenile idiopathic arthritis

^b^Connective tissue disease

^c^Auto-inflammatory diseases

^d^Disease-modifying antirheumatic drugs

At inclusion, the need for occupational therapy, hypnotherapy, psychological support and considering homeopathy were associated with decreased well-being score − 1.26 (95% CI [− 2.41; − 1.22], *p* = 0.030), − 1.29 (95% CI [− 2.22; − 0.36], *p* = 0.006), − 1.45 (95% CI [− 2.21; − 0.70], *p* < 0.001) and − 1.96 (95% CI [− 3.70; − 0.22],* p* = 0.026), respectively as was the need for adjustment of school tests − 1.55 (95% CI [− 3.04; − 0.06], *p* = 0.041).

We found no significant association between the well-being score and age, sex or pathology group.

The well-being score improved significantly, by 0.04 score units, every 6 months (95% CI [0.03; 0.06] *p* < 0.001). The evolution of the well-being score (mean) over time (every 6 months) in RESRIP patients is presented in Additional file 1: Fig. S1 to note that the number of observations decreases every 6 months (from 219 observations at inclusion to 2 observations at 72 months).

## Discussion

To the best of our knowledge, this is the first study to compare well-being in different pediatric rheumatic diseases (PRD): JIA, CTD and AIDs. The present study aimed to identify the sociodemographic/clinical characteristics, ongoing medications, needed paramedical services and educational actions associated with well-being, in pediatric patients included in RESRIP and to evaluate the evolution of well-being over time in patients benefiting from comprehensive care and daily support. Well-being was negatively associated with the need for occupational therapy, hypnotherapy or psychological support, the use of homeopathy and the need for adjustment of school tests at inclusion. It did not differ significantly between disease groups but significantly improved over time.

The average well-being scores in the three groups were in favor of average well-being. The absence of poor well-being scores (< 6) is consistent with the literature. Indeed, it has been shown that the well-being of children with chronic diseases tends to be similar to that of their peers without chronic disease [19]. Similarly, Feldmann et al. showed that cognitive performance and psychosocial coping skills of young patients with systemic JIA (SJIA) were not affected by disease activity and duration [20], supporting the hypothesis that patients successfully adapt to their chronic disease. This ability to adapt could therefore explain the relatively good score in chronically ill patients as in our population.

Interestingly, as suggested in the literature [5, 18, 19], no significant differences in well-being were found between the different disease groups. This supports the idea that well-being does not depend on the disease as such, but rather on its impact on daily life. In children with JIA, suboptimal health-related quality of life (HRQoL) has been reported up to 6 years after diagnosis, even in patients in remission or with minimal activity (5). Conversely, in patients with SJIA, mental health and psychosocial functioning improved over time [20]. But, like suboptimal HRQoL, this improvement was not correlated with disease activity. In this study, improvement was correlated with family adversity. This demonstrates that HRQoL and well-being are subjective measures that reflect not only the patient's feelings about his or her health and functional status, but more importantly, how he or she feels about participating in society.

The use of homeopathy as the need for psychological support or for hypnosis were negatively associated with the well-being score. To note that these treatments are most often suggested for patients with chronic pain and/or mood disorders such as anxiety [21–23]. Our results were in line with other studies. Anyfanti et al. showed, in adults with rheumatic diseases (360 patients aged 54 years on average with rheumatoid arthritis (30.9%), seronegative spondyloarthropathy (19.6%), osteoarthritis (18.3%), mixed connective tissue disease (11.2%), systemic lupus erythematosus (31 patients, 8.5%), and systemic sclerosis (3%)), that the factors associated with a poor quality of life were: 1/ anxiety, 2/ altered physiological functioning (functional limitation or pain) and 3/ duration of the disease [5]. The same results were reported in juvenile dermatomyositis [7].

Psychological distress, depression and anxiety were found correlated with the quality of life (QoL) of rheumatologic patients [24–26], including younger patients [27]. Uguz et al. [28] found that major depression negatively affected the QoL of people with Behçet disease and that QoL was negatively associated with the severity of depressive symptoms.

As with the need for psychological support and hypnotherapy, we found a negative impact of the need for adjustment of school tests on well-being. This finding could be explained by severe illness having had an impact on school attendance but also by the presence of chronic pain, fatigue and/or functional limitations.

Finally, we revealed an improvement in well-being over time. Although, longitudinal studies of well-being in PRD are sparse, our results were similar to the few studies conducted on JIA despite using different instruments [6, 29]. Oen et al. investigated HRQoL in a large prospective inception cohort of 1249 patients with newly diagnosed JIA, by using the Juvenile Arthritis Quality of Life Questionnaire (JAQQ; score range 1–7) and Health-related Quality of My Life (HRQoML, an instrument based on personal valuations). The authors found a gradual improvement in median JAQQ and HRQoML scores over time [6]. Listing et al. investigated 953 patients with recent-onset JIA from a prospective observational cohort in Germany and 491 healthy controls. The participants were followed for 3 years and regularly asked about their HRQoL by use of the Pediatric Quality of Life Inventory 4.0. The HRQoL of the JIA patients significantly improved during the 36 months of follow-up. The authors explained the improvement by decreased disease activity and by pain management [29].

The improvement in our patients’ well-being score may also be related to the fact that RESRIP was able to comprehensively attend to the physical and social needs of these patients. RESRIP provides a link between private and hospital care, to facilitate the daily life of families and to support the medical, paramedical and school professionals attending these patients. In particular, RESRIP has done a great deal of work in collaboration with doctors, school nurses and school directors to explain its pathologies and facilitate the success and integration of its children into school. When we look at the evolution of the score of the different items studied in the well-being score, the school life item seems to be the one that progresses the most between inclusion and the end of the study (Additional file 1: Fig. S1). In addition, the link with a nurse is probably easier to establish for a patient than the referring physician. The nurse is also more available and therefore more available to the patient and the family. Hence, the actions implemented by RESRIP may have contributed to this improvement in well-being, especially because in our population, we found no association of disease duration and well-being at inclusion. In Additional file 1: Fig. S2, we can notice a fluctuation in the mean of well-being score with time. It is important to note that the number of the observations decreased with time depending of the response rate and the duration for which the patients have been included in RESRIP. Moreover, responding patients may be those who are the most in need for RESRIP’s help.

The possible positive impact generated by the care of patients, by a health network or an equivalent structure, has been demonstrated. In addition to medical care, nurses play a key role in supporting the specialist team, especially in pediatric rheumatology [30]. They are able to recognize poor disease control and provide information on treatment and know how to access additional support. Nurses also ensure the link between the medical practitioner, other health providers, and the family. The impact of telenursing has mostly been studied in adults with chronic disease and found to decrease hospitalization rates, emergency department visits, exacerbations, number of hospitalizations, and mean duration of bed days [31, 32]. Finally, nurses were found to reduce health problems while increasing patient and family satisfaction during the management of chronic, debilitating pediatric rheumatic diseases, especially when administered in the early diagnosis period [30].

RESRIP is a pilot project. It is the first health network for patients with rare diseases. It has continued to develop since its creation at the end of 2013 and currently cares for more than 500 patients with PRD living in the Ile-de-France region. It comprises a team of 3 nurses and 2 coordinating doctors, each working part-time, as well as a secretary and a director. This health network acts in partnership with patient organizations, nurse coordinators from the centers of reference and the FAI2R rare disease health network (https://www.fai2r.org). This structure is coveted by other regions in France. We believe in the usefulness and necessity of this type of structure for better care of rare diseases in general for there is no equivalent structure allowing this city-hospital link and this daily support in rare diseases. Indeed, the difficulty for these families or the professionals who surround them (GP, physiotherapists, school professionals, etc.) is linked to the lack of knowledge of these different pathologies. This makes it impossible to use the usual networks in the city, especially general practitioners, as is the case for frequent chronic diseases such as diabetes or asthma, for example.

Our study has several limitations. First, the questionnaire was completed by the patients or their parents and the responses where then analyzed together, regardless of who completed the questionnaires. Although this maximizes the total sample size, there may be discrepancies between child and proxy perceptions, especially for older children [33–35]. Unfortunately, proxy assessments are unavoidable for young children for whom the concept of quality of life may not be easy to understand. Lundberg and Eriksson revealed that parent–child differences in QoL evaluation depend on the HRQoL instrument used [36]. Parents seemed more likely to perceive their child with a diagnosis of JIA as more vulnerable than parents of a healthy child or a child with other chronic health conditions. The child with JIA was also considered more vulnerable by the parents if functionally disabled and with short disease duration [37]. Parents of a child with short disease duration, with intense reported pain, taking second-line drug therapy, or with functional impairment tended to report high disease activity [38]. Hence, our results might be biased in case of parent–child differences in well-being assessment. Second, comparing our well-being scores to those of other studies was not possible because of the differences in measurement instruments, especially because our instrument is not validated and lacks a known cutoff point, although the Cronbach’s alpha was substantial (0.77). However, this study brings attention to the psychosocial issues which are not taken into consideration when using validated tools such as Childhood Health Assessment Questionnaire (CHAQ). The latter measures physical disease status only and therefore captures only half the picture of how children/adolescent with CIR disease are really doing. Furthermore, our measurements may be subject to recall bias because participants were asked to report their well-being during the last 6 months. Moreover, for the follow-up of patients every 6 months, some questionnaires were self-reported and therefore sometimes misinterpreted or not returned, which may imply information bias. Third, we did not use a control group to estimate cause and effect. Fourth, other unmeasured confounders may be associated with the well-being of children with CRD such as chronic pain. Finally, the results of our study cannot be generalized to all children with JIA, CTD or AIDs living in the Ile-de-France region because of selection bias.

Despite these limitations, our study has certain strengths. This is, to the best of our knowledge, the first longitudinal study conducted in the Ile-de-France region that evaluated the well-being in PRD.

In conclusion, well-being seems to be more associated with the impact of CIR disease than the type of disease itself. Chronic pain, psychological problems or functional failure are the factors with the greatest impact on quality of life, as described in the literature. Importantly, we demonstrate a significant improvement in well-being over time in RESRIP patients. These results provide a better understanding of a patient's condition in the course of a chronic disease and underline the importance of comprehensive patient management and thus the usefulness of care networks such as RESRIP.

## Supplementary Information

**Additional file 1**. **Figure S1**: Evolution of the school well-being score over time. **Figure S2**: The mean of well-being scores over time (n=406 and a total of 728 observations).

**Additional file 2.** Questionnaire created by RESRIP to assess the well-being of patients, to be completed by the parents and/or the child depending on age.

## Data Availability

“Please contact author for data requests.”
