# Supplementary material for: Well-being in chronic pediatric inflammatory rheumatic diseases: the experience of a French healthcare network
Source: Orphanet J Rare Dis. 2023 Mar 7;18:46. doi: 10.1186/s13023-023-02655-z (PMC9990204; doi:10.1186/s13023-023-02655-z)
Supplement: Supplementary file 1 — Additional file 1. Figure S1: Evolution of the school well-being score over time. Figure S2: The mean of well-being scores over time (n=406 and a total of 728 observations). [file 13023_2023_2655_MOESM1_ESM.docx]

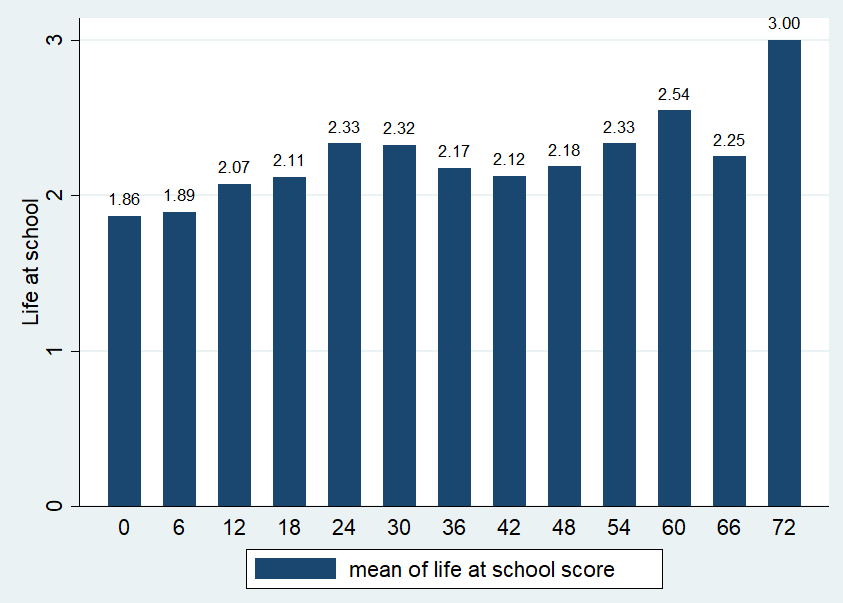


Supplemental Figure 1: Evolution of the school well-being score over time

Supplemental Figure 2: The mean of well-being scores over time (n=406 and a total of 728 observations).
