# Supplementary material for: Well-being in chronic pediatric inflammatory rheumatic diseases: the experience of a French healthcare network
Source: Orphanet J Rare Dis. 2023 Mar 7;18:46. doi: 10.1186/s13023-023-02655-z (PMC9990204; doi:10.1186/s13023-023-02655-z)
Supplement: Supplementary file 2 — Additional file 2. Questionnaire created by RESRIP to assess the well-being of patients, to be completed by the parents and/or the child depending on age. [file 13023_2023_2655_MOESM2_ESM.docx]

**Follow-up assessment: assessment to be completed by parents for children aged < 7 years**

These 4 questions are about what has happened in **the last 4 weeks**. For each question, give the answer that is closest to what you think your child has felt.

**Q1: Has your child had any pain? If so, how much pain would you rate on a scale of 0-10 (0 being no pain and 10 being maximum pain)?**

| **0** | **1** | **2** | **3** | **4** | **5** | **6** | **7** | **8** | **9** | **10** |
| --- | --- | --- | --- | --- | --- | --- | --- | --- | --- | --- |
|  |  |  |  |  |  |  |  |  |  |  |

**Q2: Has your child been tired? If so, how would you describe his/her fatigue on a daily basis? Is it :**

- Absent (he/she is not tired),
- Low (he/she is a little tired but can do everything)
- Moderate (fatigue sometimes prevents him/her from doing certain activities or going to school)
- Severe (prevents him/her from doing activities or going to school)

**Q3: Has your child experienced night wakings? If yes, how many on average?**

0 󠅪󠅪 1 󠅪󠅪 2 󠅪󠅪 3 󠅪󠅪 More than 3 󠅪󠅪

**Q4: Does your child's illness have an impact on everyday life? If so, how much do you estimate the maximum impact on a scale of 0-10 (0 being no impact and 10 being a major impact)?**

| **0** | **1** | **2** | **3** | **4** | **5** | **6** | **7** | **8** | **9** | **10** |
| --- | --- | --- | --- | --- | --- | --- | --- | --- | --- | --- |
|  |  |  |  |  |  |  |  |  |  |  |

This questionnaire is about what has happened **in the last 6 months**. For each question, give the answer that is closest to how you felt.

**In general, would you say that:**

His/Her physical health is:

Very satisfactory 󠅪 󠅪󠅪 Satisfactory ⁭󠅪 Average 󠅪 󠅪󠅪 Poor 󠅪󠅪 DK* 󠅪󠅪

His/Her family life is:

Very satisfactory 󠅪 󠅪󠅪 Satisfactory ⁭󠅪 Average 󠅪 󠅪󠅪 Poor 󠅪󠅪 DK* 󠅪󠅪

His/Her life with his/her friends is:

Very satisfactory 󠅪 󠅪󠅪 Satisfactory ⁭󠅪 Average 󠅪 󠅪󠅪 Poor 󠅪󠅪 DK* 󠅪󠅪

His/Her school life is:

Very satisfactory 󠅪 󠅪󠅪 Satisfactory ⁭󠅪 Average 󠅪 󠅪󠅪 Poor 󠅪󠅪 DK* 󠅪󠅪

*DK: do not know

In the last 6 months, how many days of absence from school/work have you had because of the illness: (pain, fever, consultations, Hospitalizations...)

For the parents: .................. Not concerned

For the child: ...................... Not concerned

**Follow-up report to be completed by children ≥ 7 years old with parental help if needed**

These 4 questions are about what has happened over the last 4 weeks. For each question, give the answer that is closest to how you felt.

**Q1: Have you had any pain? These faces show how much pain you can have. These faces show someone in increasing pain (no pain at all to very, very bad). Show me the face that shows how much pain you are in right now.**


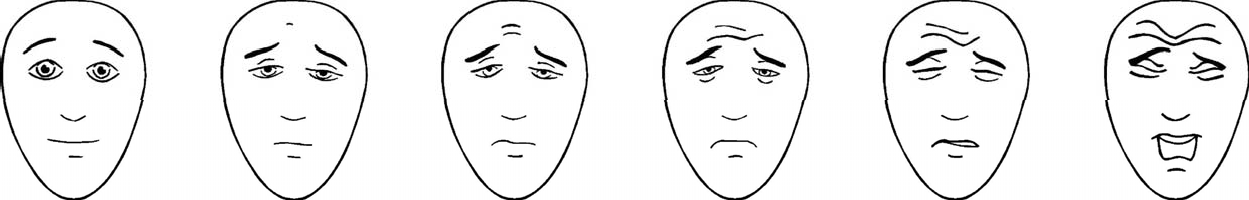


󠅪󠅪 󠅪󠅪 󠅪󠅪 󠅪󠅪 󠅪󠅪 󠅪󠅪

**Q2: Have you been tired in the last 4 weeks? If so, how would you describe your day-to-day fatigue? Is it :**

- Absent (you are not tired)
- Low (you are a little tired but you can do everything)
- Moderate (fatigue sometimes prevents you from doing certain activities or going to school)
- Severe (prevents you from doing your activities or going to school)

**Q3: How many times have you woken up at night because of your illness in the last 4 weeks (average) :**

0 󠅪󠅪 1 󠅪󠅪 2 󠅪󠅪 3 󠅪󠅪 More than 3 󠅪󠅪

**Q4: Does your illness have an impact on your everyday life (friends, school, activities...)?**

**If yes, please indicate:**

Not at all 󠅪󠅪 A little 󠅪󠅪 A lot 󠅪󠅪 Very much 󠅪󠅪

This questionnaire is about what has happened in **the last 6 months**. For each question, give the answer that is closest to how you felt.

**In general, would you say that :**

Your physical health (your body) is :

Very satisfactory 󠅪 󠅪󠅪 Satisfactory ⁭󠅪 Average 󠅪 󠅪󠅪 Poor 󠅪󠅪 DK* 󠅪󠅪

Your family life is :

Very satisfactory 󠅪 󠅪󠅪 Satisfactory ⁭󠅪 Average 󠅪 󠅪󠅪 Poor 󠅪󠅪 DK* 󠅪󠅪

Your life with your friends is :

Very satisfactory 󠅪 󠅪󠅪 Satisfactory ⁭󠅪 Average 󠅪 󠅪󠅪 Poor 󠅪󠅪 DK* 󠅪󠅪

Your life at school is :

Very satisfactory 󠅪 󠅪󠅪 Satisfactory 󠅪󠅪 Average 󠅪 󠅪󠅪 Poor 󠅪󠅪 DK* 󠅪󠅪

*DK: do not know

In the last 6 months, how many days of absence from school/work have you had because of the illness: (pain, fever, consultations, HDJ...)

- For the parents: .................. Not concerned
- For the child: ...................... Not concerned
